# Age and HPV type as risk factors for HPV persistence after loop excision in patients with high grade cervical lesions: an observational study

**DOI:** 10.1186/s12893-016-0185-7

**Published:** 2016-10-06

**Authors:** Laurențiu Pirtea, Dorin Grigoraş, Petru Matusz, Marilena Pirtea, Lavinia Moleriu, Anca Tudor, Răzvan Ilina, Cristina Secoşan, Florin Horhat, Octavian Mazilu

**Affiliations:** 1 Department of Obstetrics and Gynecology, University of Medicine and Pharmacy “Victor Babeş”, Timişoara, Romania; 2 Department of Anatomy, University of Medicine and Pharmacy “Victor Babeş”, Timişoara, Romania; 3 Department of Obstetrics and Gynecology, County Hospital Timişoara, Timișoara, Romania; 4 Department of Informatics and Biostatistics, University of Medicine and Pharmacy “Victor Babeş”, Timişoara, Romania; 5 Department of Surgery, University of Medicine and Pharmacy “Victor Babeş”, str. Dimitrie Cantemir, nr. 1, Zip Code 300001 Timişoara, Romania; 6 Department of Microbiology, University of Medicine and Pharmacy “Victor Babeş”, Timişoara, Romania; 7 County Hospital Timișoara, Hector street, number 1, Timișoara, Romania

**Keywords:** HPV type 16, Age, Infection persistence, LEEP

## Abstract

**Background:**

Persistent infections with high risk human papillomaviruses (HR-HPV) cause virtually all cervical cancers.

**Methods:**

An observational study was conducted aiming to estimate the rate of HPV infection persistence after LEEP in patients with high grade squamous intraepithelial lesions (HSIL). Moreover, the study investigated if persistence is age related. For this reason a total of 110 patients were included between January 2010 and June 2015.

**Results:**

At 6 months after LEEP the overall HPV infection persistence rate was 40.9 %, at 12 months 20 % and at 18 months 11.8 %. Type 16 showed the highest persistence rate: 27.3 % at 6 months, 12.7 % at 12 months and 10 % at 18 months after LEEP. The persistence for HPV type 16 at 6 months after LEEP was significantly higher in the group > =36.5 years old compared to the persistence rate in the group <36.5 years old (*p* = 0.0027, RR = 2.75, 95 %ϵ(1.34; 5.64)) (see Table 3).

**Conclusions:**

LEEP does not completely eradicate HPV infection. HPV persistence rate after LEEP is higher in infections with type 16 and in women older than 36.5 years.

**Electronic supplementary material:**

The online version of this article (doi:10.1186/s12893-016-0185-7) contains supplementary material, which is available to authorized users.

## Background

Virtually all cervical cancers are caused by persistent high-risk human papillomaviruses (HR-HPV) infection [1–3]. One of the indirect factors for HPV persistence is considered to be the old age [4]. Genital HPV infection is presumably the most prevalent sexually transmitted infection [4]. A particularly high risk for the acquisition of HPV infection is described in young women, soon after they become sexually active [5].

The prevalence of HPV infection in women older than 30 is significantly lower than that described in younger women at the mean age of the first sexual intercourse. Despite the fact that these infections are mostly controlled or self-limited by the immune system, the determinants of age-specific prevalence variation in older women remain uncertain [6]. It is considered that the clearance of the infection is immune-mediated and mostly type-specific.

The management of women with cervical intraepithelial neoplasia (CIN) is crucial, given that improper management may increase the risk of developing cervical cancer, whereas overtreatment increases the risk of complications related to preterm delivery or other. Therefore, appropriate management is essential in terms of cancer prevention [7, 8].

The standard procedure for conservative management of high-grade CIN is large loop excision of the transformation zone (LEEP) or conization. Although this treatment is generally sufficient, resulting in a complete cure, Arbyn et al [9] reported an average of 10 % residual or recurrent disease in the treated cases.

Some HPV infections may persist, despite the relatively high post-conization HPV clearance rate [10]. A number of risk factors for residual or recurrent disease have been identified in the treatment of CIN lesions. According to Nam and Heymans, these are: age, menopause status, cytology grade, margin involvement and HPV viral load [10, 11].

The objectives of our study were to estimate the rate of HPV infection persistence after LEEP in patients with high grade squamous intraepithelial lesions (HSIL) and to investigate if persistence is age related.

In Romania cervical cancer is the first leading cause of cancer deaths in women aged 15 to 44 years, with over 4300 new cases diagnosed each year. Infection with HR HPV types was found in 86.8 % of the cases and the prevalence HPV type 16 is 45 % among women with high grade lesions [12]. A national program that started in 2012 for the screening and prevention of cervical cancer is still ongoing in Romania. In addition, all women aged over 16 can benefit from a free Pap smear. Moreover, the cases with cytological abnormalities are referred to specialized centers.

## Methods

Patient selection: We performed an observational study. We included in the study all patients with HSIL cytology on PAP smear that had no previous treatment for cervical lessions, who were referred for LEEP to the department of obstetrics and gynecology of the University of Medicine and Pharmacy “Victor Babeș” Timișoara, between January 2010 and June 2015. Patients were first evaluated trough the national screening program. Those with cytological abnormalities were referred to our center. Another Pap smear was performed and patients with HSIL were referred to our department. All patients referred for LEEP had Pap smears interpreted by the same pathology team. Conventional cytology was performed and evaluated according to the criteria of Bethesda 2001. All patients were evaluated by colposcopy and International Federation for Cervical Pathology and Colposcopy (IFCPC) criteria were used. All patients underwent LEEP under colposcopic vision after Lugol solution application. With the procedure all colposcopically abnormal findings were excised, aiming for a tissue depth of at least 6 mm. Every procedure was performed by the same team of surgeons. HPV DNA testing was performed before LEEP in all cases and repeated during the follow up visits at 6, 12 and 18 months after surgery. The investigated outcome was HPV persistence on HPV DNA test at 6, 12 and 18 months after LEEP. Patients who were negative for HPV DNA before LEEP were excluded from the study. All samples were examined using LINEAR ARRAY HPV Genotyping Test (CE-IVD), based on reverse hybridization of amplicons. The DNA of 37 HPV types (6, 11, 16, 18, 26, 31, 33, 35, 39, 40, 42, 45, 51, 52, 53, 54, 55, 56, 58, 59, 61, 62, 64, 66, 67, 68, 69, 70, 71, 72, 73, 81, 82, 83, 84, IS39 and CP6108) was detected in cervical samples by multiplex PCR targeted to the conserved L1 region of the viral genome. The Gene Amp PCR System 9700 was used for genotyping test according to the manufacturer’s instructions. Automated hybridization and detection of HPV DNA was done on the ProfiBlot 48 (Tecan Trading AG, Zurich, Switzerland).

All specimens were sent to histopathological exam. Patients with positive resection margins after LEEP were excluded from the study.

Follow up visits were scheduled at 6, 12 and 18 months after surgery. DNA HPV testing was performed for each patient at each visit.

Informed consent was obtained from every patient prior to their inclusion in the study. All procedures have been performed in accordance with the ethical standards laid down in the 1964 Declaration of Helsinki and its later amendments and were approved by the Institutional Review Board and Ethical Committee of “Victor Babeş” University of Medicine and Pharmacy Timişoara – reference number of ethics approval 20/2010.

Statistical analysis was conducted using the following software SPSS v17, Epi Info 7 and Microsoft Excel. For this data we computed a descriptive statistics, we used parametrical statistical tests (Z test for proportion) and we performed a risk analysis using a chi square test and risk indicators. The cutoff point was considered the median age in our group. The Z test for proportion was computed in order to measure the persistence rate of HPV types. We performed a risk analysis considering as an exposure the age above the median age in our group.

## Results

A total of 129 patients were referred to our clinic with HSIL on PAP Smear test. Positive margins after LEEP were found in 7 patients and they were excluded from the study. The HPV test was negative in 12 patients with HSIL and they were also excluded. The remaining 110 patients were followed up. Data collection was complete for all 110 patients at each time point and the study visits were balanced individually for every patient at 6 months apart. The HPV types detected at the start of the trial were 49.1 % type 16, 21.8 % type 18, 20 % type 31, 26.4 % type 33, 10.9 % type 35, 6.4 % type 45, 30 % type 52, 9.1 % type 58, 7.3 % type 6, 4.5 % type 11 and 4.5 % other types. The co-infection with multiple HR HPV types was found in 68.2 % of our patients. At 6 months after LEEP the overall persistence was 40.9 % (45 patients), at 12 months 20 % (22 patients) and at 18 months 11.8 % (13 patients). The rate of persistence in our group at 6, 12 and 18 months for each HPV type and the distribution of HPV types (including patients with multiple types) found before LEEP and the distribution of HPV types persistent at 6, 12 and 18 months are shown in Table 1.

**Table 1:** Frequency table for all HPV types

HPV Types	Before LEEP	6 months	12 months	18 months
16	54 (49.1 %)	30 (27.3 %)	14 (12.7 %)	11 (10.0 %)
18	24 (21.8 %)	5 (4.5 %)	1 (0.9 %)	0 (0 %)
31	22 (20 %)	2 (1.8 %)	0 (0 %)	0 (0 %)
33	29 (26.4 %)	4 (3.6 %)	1 (0.9 %)	0 (0 %)
35	12 (10.9 %)	0 (0 %)	0 (0 %)	0 (0 %)
45	7 (6.4 %)	0 (0 %)	0 (0 %)	0 (0 %)
52	33 (30.0 %)	9 (8.2 %)	4 (3.6 %)	0 (0 %)
58	10 (9.1 %)	2 (1.8 %)	1 (0.9 %)	0 (0 %)
6	8 (7.3 %)	2 (1.8 %)	2 (1.8 %)	2 (1.8 %)
11	5 (4.5 %)	1 (0.9 %)	1 (0.9 %)	1 (0.9 %)
other types	5 (4.5 %)	0 (0 %)	0 (0 %)	0 (0 %)
coinfections	75 (68.2 %)	10 (9.1 %)	2 (1.8 %)	1 (0.9 %)
total number of patients with HPV infection	110 (100.0 %)	45 (40.9 %)	22 (20.0 %)	13 (11.8 %)

Frequency table for all HPV type - before LEEP, at 6 months, 12 months and 18 months

The HPV clearance rate (proportion) after LEEP is statistical significant at 6 and 12 months. The overall HPV persistence rate was lower at 18 months compared to 12 months, but the difference was not statistically significant (Table 2).

**Table 2:** The evolution in time for infections

Moments of time		Type 16 HPV		Type 16 + other HPV types		Co-infections	
		*p* value significance	Level of significance	*p* value significance	Level of significance	*p* value significance	Level of significance
Before LEEP	6 months	0001^s^	0,01	<0001^s^	0001	<0001^s^	0001
	12 months	<0001^s^	0001	<0001^s^	0,001	<0,001^s^	0,001
	18 months	<0,001^s^	0,001	<0,001^s^	0,001	<0,001^s^	0,001
6 months	12 months	0,011^s^	0,05	0,340^ns^	0,05	0,037^s^	0,05
	18 months	0,002^s^	0,01	0,249^ns^	0,05	0,013^s^	0,05
12 months	18 months	0,675^ns^	0,05	0,824^ns^	0,05	0,995^ns^	0,05

The evolution in time for infections: HPV 16, HPV 16 and at least another HPV type and for co-infections (^s^ – significant difference, ^ns^ - insignificant difference)

**Fig. 1:**
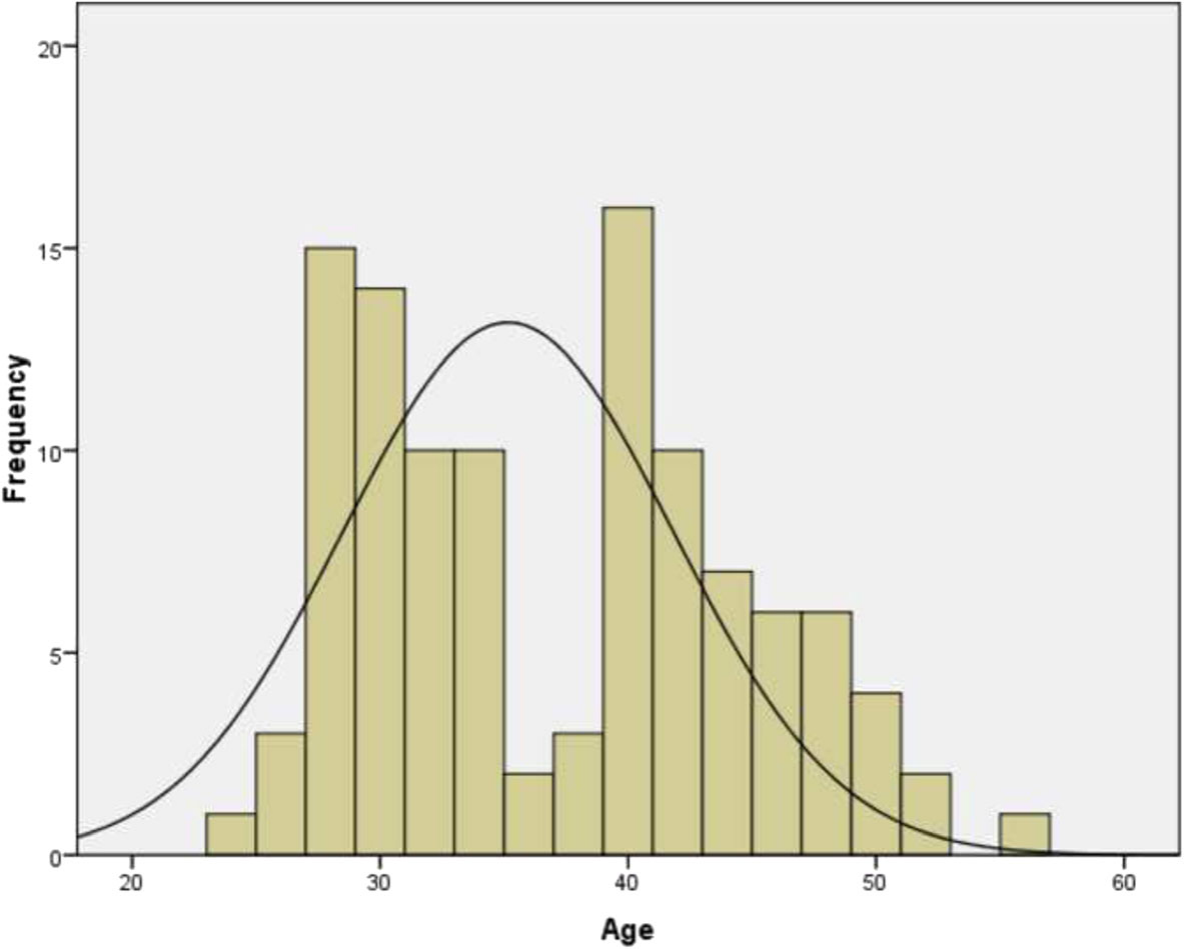
The age distribution histogram – the median age is 36.5 years. This graphic shows patient distribution according to the age of the patients

The age distribution is shown in Fig. 1 and Table 3. The median age in our group was 36.5 years. We found a higher persistence rate for HPV16 in patients who were older than 36.5 years and in patients presenting co-infection (by co-infection we mean infection with at least two different HPV types). We performed a comparative analysis for the HPV type 16 infection rate before LEEP and at each follow up visit and we found that the clearance rate in the group > =36.5 years was significantly lower in the first 6 months (*p* = 0.0027, RR = 2.75, 95 %ϵ(1.34; 5.64)). All the results are presented in Table 4. For co-infections we performed the same analysis and we found that age over 36.5 years was associated with a higher persistence rate during the first 12 months (*p* = 0.01, RR = 2.67, 95 %ϵ(1.13; 6.30)). All of these results are shown in Table 5.

**Table 3:** The age distribution

Age	Frequency	Percent	Age	Frequency	Percent
24	1	0,91	40	7	6,36
26	3	2,73	41	5	4,55
27	5	4,55	42	5	4,55
28	10	9,09	43	1	0,91
29	8	7,27	44	6	5,45
30	6	5,45	45	2	1,82
31	5	4,55	46	4	3,64
32	5	4,55	47	4	3,64
33	7	6,36	48	2	1,82
34	3	2,73	49	3	2,73
36	2	1,82	50	1	0,91
37	3	2,73	52	2	1,82
39	9	8,18	55	1	0,91

The age distribution, the age frequency and the corresponding percentage for our group

**Table 4:** Risk analysis for the persistence of HPV 16

Volume *N* = 110	HPV 16+	HPV 16-	Total	*p*-value Risk analysis
6 Months				
> = 36.5	22	33	55	*p* = 0.0027
<36.5	8	47	55	RR = 2.75 95 %ϵ(1.34; 5.64)
Total	30	80	110	
12 Months				
> = 36.5	10	45	55	*p* = 0.0861
<36.5	4	51	55	RR = 2.5 95 %ϵ(0.83; 7.49)
Total	14	96	110	
18 Months				
> = 36.5	8	47	55	*p* = 0.1120
<36.5	3	52	55	RR = 2.67 95 %ϵ(0.75; 9.53)
Total	11	99	110	

Risk analysis for the persistence of HPV 16 (the contingence tables, the *p* values, the relative risk values and the risk inference intervals for 95 % of the population). With HPV16+ we marked the patients having HPV16, and with HPV16- the patients without this HPV type. We considered as an exposure the age above 36.5 years

**Table 5:** Risk analysis for the persistence of HPV coinfections

Volume *N* = 110	HPV+	HPV-	Total	*p*-value Risk analysis
6 Months				
> = 36.5	32	23	55	*p* < 0.001
<36.5	13	42	55	RR = 2.46 95 %ϵ(1.45; 4.16)
Total	45	65	110	
12 Months				
> = 36.5	16	39	55	*p* = 0.0171
<36.5	6	49	55	RR = 2.67 95 %ϵ(1.13; 6.30)
Total	22	88	110	
18 Months				
> = 36.5	8	47	55	*p* = 0.3755
<36.5	5	50	55	RR = 1.60 95 %ϵ(0.55; 4.58)
Total	13	97	110	

Risk analysis for the persistence of HPV coinfections (the contingence tables, the *p* values, the relative risk values and the risk inference intervals for 95 % of the population). We marked with co-infections (HPV+) the patients who have at least two different HPV types and with HPV- patients who have just one HPV type. We considered as an exposure the age above 36.5 years

We found no significant differences between the persistence rate for co-infections including type 16 versus infection with type 16 alone. The results are shown in Table 6.

**Table 6:** Risk analysis for the persistence of HPV 16 with other HPV types and only HPV16

Volume	HPV 16+ other HPV types	HPV 16	Total	*p*-value Risk analysis
6 Months				
> = 36.5	7	15	22	*p* = 0.77
<36.5	3	5	8	RR = 0.85 95 %ϵ(0.28; 2.51)
Total	10	20	30	
12 Months				
> = 36.5	0	10	10	*p* = 0.019
<36.5	2	2	4	RR = 0 undefined
Total	2	12	14	
18 Months				
> = 36.5	0	8	8	*p* = 0.102
<36.5	1	2	3	RR = 0 undefined
Total	1	10	11	

Risk analysis for the persistence of HPV 16 with other HPV types and only HPV16 (the contingence tables, the *p* values, the relative risk values and the risk inference intervals for 95 % of the population). We considered as an exposure the age above 36.5 years

## Discussion

Despite the removal of the entire lesion by cone excision, with negative margins, the HPV infection can persist in some cases. Studies investigating the clearance/persistence of HPV infection after LEEP have reported that age, lesion grade, and margin status are risk factors for HPV persistence.

Since the presence of positive margins is considered a major factor for HPV persistence and disease recurrence and progression, we excluded patients with positive margins after resection from our study, as we wanted to investigate the persistence of HPV infection in patients with negative margins.

Although LEEP does not completely eradicate HPV infection, our results indicate that most HR-HPV infections are cleared after LEEP with negative margins. The clearance rate is increasing gradually after surgery. Our persistence rate was 40.9 % at 6 months, 20 % at 12 months and 11.8 % at 18 months. We identified a persistence rate higher than the one reported by other authors: Kim et al [13] reported a persistence rate of 14.3 %, 2.2 % and 1.1 % at 6, 12 and 18 months. High persistence rates, similar to ours, were found only by Song et al [14], who reported a persistence rate of 43.8 % at 6 months in patients with high viral load before LEEP [14]. We consider that our criteria for patient selection and the fact that only patients with HSIL were included is the cause for our high persistence rate.

Our results indicate that HPV type 16 has the lowest clearance rate. Kim et al [13], Heymans et al [11] and Nam et al [10] also found that HPV type 16 is a factor for infection persistence after treatment. Therefore, patients with HPV type 16 should be carefully monitored after LEEP [10, 11, 13].

The value of age as a factor that favors HPV persistence after LEEP is a subject of controversy. Costa et al 2003 and Sarian et al 2004 found that women older than 35 years old had a significantly higher risk for HPV persistence after LEEP [15, 16]. On the other hand, more recent studies performed by Nam et al 2009 and Park et al [17] found no correlation between age of patient and HPN infection persistence after LEEP [10, 17]. Our results indicate that age is a risk factor for the persistence after conization only for HPV type 16. At the end of our study the persistence for HPV16 was 7.3 % for the group > =36.5 years old and 2.7 % for the group <36.5 years old (with *p* = 0.1120, RR = 2.67, 95 %ϵ(0.75; 9.53)). In the first 6 months after LEEP we have significant differences between this two group ages (*p* = 0.0027, RR = 2.75, 95 %ϵ(1.34; 5.64)).

We consider this information valuable, as HPV type 16 seems to have the highest pathogenicity. We did not find in literature data about age as a risk factor for the persistence of HPV type 16 alone. For that reason, we consider that this adds value to our study.

The value of age as a predictor for disease recurrence is also subject for debate: Verguts et al 2006 found higher age at LEEP is associated with higher rate of disease recurrence, while Ryu et al 2012 found no correlation between age and disease recurrence [18, 19]. Since most recurrences are associated with the persistence of HPV type 16, we consider that women with HPV type 16 and older than 36.5 years should be closely followed.

In our study group we identified a high percentage (68 %) of co-infection with multiple HPV types. According to the findings of Jaisamrarn et al [20], concomitant HPV infection increase the risk of progression to a lesion, suggesting that multiple HPV infections could influence disease progression. We consider that our high rate of patients co-infected with multiple HPV types is due to the selection of patients with HSIL only [20].

The limitation of our study is that we tested for HR-HPV only patients with HSIL and this artificially increases the percentage of HR- HPV positive patients.

The strengths of the study are represented by the nature of the study and the fact that only patients with HSIL were selected. This way we investigated the very category of patients that are likely to be infected with HR-HPV and that are exposed to recurrence after LEEP and disease progression to cancer.

## Conclusions

Although LEEP does not completely eradicate HPV infection, our results indicate that most HR-HPV infections are cleared after LEEP with negative margins.

Moreover, HPV type 16 and age over 36.5 years are factors that favor infection persistence.

## Additional file

Additional file 1: Datasets. (XLS 106 kb)

## Abbreviations

CIN: Cervical intraepithelial neoplasia
HPV: Human papilloma virus
HR-HPV: High-risk human papilloma virus
HSIL: High grade squamous intraepithelial lesions
IFCPC: International Federation for Cervical Pathology and Colposcopy
LEEP: Loop excision of the transformation zone
